# Impacts of Chemerin Levels and Antioxidant Capacity on the Severity of Cardiovascular Autonomic Neuropathy in Patients with Type 2 Diabetes and Prediabetes

**DOI:** 10.3390/biomedicines11113024

**Published:** 2023-11-10

**Authors:** Yun-Ru Lai, Chih-Cheng Huang, Ben-Chung Cheng, Wen-Chan Chiu, Ting-Yin Lin, Hui-Ching Chiang, Chun-En Kuo, Cheng-Hsien Lu

**Affiliations:** 1 Departments of Neurology, Kaohsiung Chang Gung Memorial Hospital, Chang Gung University College of Medicine, No. 123, Ta Pei Road, Niao Sung Hsiang, Kaohsiung City 83301, Taiwan; yunrulai@cgmh.org.tw (Y.-R.L.); hjc2828@gmail.com (C.-C.H.); a0917604453@gmail.com (H.-C.C.); 2 Departments of Hyperbaric Oxygen Therapy Center, Kaohsiung Chang Gung Memorial Hospital, Chang Gung University College of Medicine, No. 123, Ta Pei Road, Niao Sung Hsiang, Kaohsiung City 83301, Taiwan; 3 Departments of Internal Medicine, Kaohsiung Chang Gung Memorial Hospital, Chang Gung University College of Medicine, No. 123, Ta Pei Road, Niao Sung Hsiang, Kaohsiung City 83301, Taiwan; benzmcl@gmail.com (B.-C.C.); testercwt@gmail.com (W.-C.C.); 4 Departments of Nursing, Kaohsiung Chang Gung Memorial Hospital, Chang Gung University College of Medicine, No. 123, Ta Pei Road, Niao Sung Hsiang, Kaohsiung City 83301, Taiwan; s971078@cgmh.org.tw; 5 Departments of Chinese Medicine, Kaohsiung Chang Gung Memorial Hospital, Chang Gung University College of Medicine, Kaohsiung City 83301, Taiwan; lecherries@gmail.com; 6 Department of Biological Science, National Sun Yat-Sen University, Kaohsiung City 80424, Taiwan; 7 Department of Neurology, Xiamen Chang Gung Memorial Hospital, Xiamen 361126, China

**Keywords:** antioxidant capacity, cardiovascular autonomic neuropathy, chemerin, composite autonomic scoring scale, type 2 diabetes and prediabetes, thiol

## Abstract

Existing evidence supports an association between chemerin levels and cardiovascular risk, while reduced thiol levels are linked to diabetes mellitus. It is hypothesized that chemerin may contribute to autonomic dysfunction and cardiovascular risk in type 2 diabetes mellitus (T2DM), potentially mediated by the antioxidant capacity of patients with well-controlled T2DM and prediabetes. Comprehensive cardiovascular autonomic testing and biomarker assessments were conducted for all participants. The severity of cardiovascular autonomic neuropathy (CAN) was evaluated using the composite autonomic scoring scale (CASS). A mediation model was employed to explore the potential relationships among chemerin levels, antioxidant capacity (indicated by thiol levels), and CAN severity (indicated by CASS values). A total of 184 participants were enrolled in this study, comprising 143 individuals with T2DM and 40 individuals with prediabetes. The findings reveal a significant negative association between thiols levels (r = −0.38, *p* < 0.0001) and the CASS values, while a positive association is observed between chemerin levels (r = 0.47, *p* < 0.0001) and the CASS values. Linear regression analysis identified chemerin and thiols as independent variables significantly associated with CASS values. Subsequent mediation analysis elucidated that thiols levels act as mediators in the relationship between elevated chemerin levels and an increased CASS value. This study shows that poor cardiovascular function, higher chemerin levels, and reduced antioxidant capacity coexist in individuals with T2DM and prediabetes. Mediation analysis suggests a pathophysiological link between high chemerin levels and low antioxidant capacity, adversely impacting CAN severity.

## 1. Introduction

Cardiovascular autonomic neuropathy (CAN) is distinguished by dysregulations within both the parasympathetic and sympathetic nervous systems. In individuals affected by type 2 diabetes mellitus (T2DM) and prediabetes, CAN shows a robust association with a diverse spectrum of cardiovascular diseases (CVDs), consequently augmenting the susceptibility to morbidity and mortality [1,2]. The pathophysiological underpinnings of cardiovascular autonomic neuropathy (CAN) are multifaceted, with compelling evidence indicating its potential to precede the onset of diabetes mellitus (DM) [3]. This emphasizes the necessity of ongoing monitoring and comprehensive management of CAN, highlighting the significance of formulating risk stratification approaches for CAN in individuals afflicted with prediabetes and T2DM.

The prevalence of CAN exhibits variability contingent upon the specific definitions and diagnostic criteria employed [4]. In accordance with the Toronto Consensus, the recommended gold standard for diagnosing CAN in individuals with T2DM comprises a comprehensive assessment encompassing seven distinct tests [5]. The composite autonomic scoring scale (CASS), introduced by Phillip Low, serves as a laboratory-based tool for quantifying CAN severity [6].

Currently, adipose tissue is recognized as a multifaceted and dynamic endocrine organ situated within the human body. It functions not only as a repository for energy but also as a regulator of energy homeostasis, cellular reactions, and metabolic balance [7].

Adipocytes, being metabolically active and possessing a pronounced secretory capacity, proficiently release a multitude of adipocytokines. They play a multifaceted role in the regulation of diverse physiological processes, encompassing appetite modulation, the orchestration of inflammatory and immune responses, glucose and lipid metabolism, the maintenance of long-term energy equilibrium, the augmentation of insulin sensitivity in insulin-responsive tissues, the preservation of cardiovascular homeostasis, and the facilitation of reproductive functions, among a spectrum of pivotal biological and physiological functions [8].

Chemerin, an adipokine primarily expressed in adipose tissue, plays a pivotal role in adipocyte differentiation, development, and the modulation of glucose and lipid metabolism [9]. Elevated concentrations of chemerin have been documented in individuals affected by obesity, those in prediabetic states, and obese individuals with T2DM [10]. Conversely, thiols, which are characterized by the presence of sulfhydryl groups, fulfill a significant function in the attenuation of oxidative stress by combating reactive oxygen species. Diminished thiol levels have been implicated in the context of diabetes mellitus [11].

While various studies have elucidated the perturbation of chemerin regulation attributable to augmented adipose tissue, potentially playing a role in the pathogenesis of cardiovascular diseases [9,12], the existing literature thus far lacks comprehensive investigation of the association between quantifying chemerin levels, oxidative stress, and the severity of CAN in patients afflicted with T2DM and prediabetes.

In line with our formulated hypotheses, a reduction in antioxidant capacity is postulated to be linked to the upregulation of chemerin expression, consequently contributing to a deterioration in CAN. The outcomes of our study hold potential significance for the advancement of therapeutic interventions in individuals with DM and have the potential to enhance the quality of life of those afflicted with T2DM and prediabetes.

## 2. Patients and Methods

**Figure 1:**
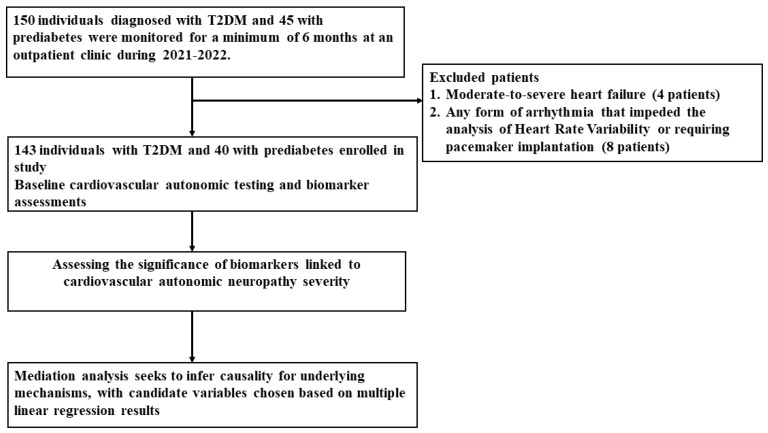
Enrollment of patients.

An observational prospective study was conducted at a tertiary medical center and a primary referral hospital with the objective of evaluating autonomic function in individuals diagnosed with T2DM and prediabetes [13]. This study involved the examination of a cohort comprising 195 patients who were assessed consecutively, including 150 individuals diagnosed with T2DM and 45 with prediabetes, during the period spanning from January 2021 to December 2022, with the enrollment commencing from the first patient and concluding with the last patient from the neurology outpatient clinic. All participants underwent continuous evaluation and were subject to regular follow-up for a minimum duration of 6 months. Exclusion criteria were applied to individuals meeting the following conditions: (1) experiencing moderate-to-severe heart failure (classified as New York Heart Association class III and IV) among 4 patients, and (2) possessing any form of arrhythmia that impeded the analysis of heart rate variability (HRV) or requiring pacemaker implantation for any reason in 8 other patients (Figure 1). Consequently, the final study cohort comprised a total of 183 patients, including 143 individuals diagnosed with T2DM and 40 individuals diagnosed with prediabetes (Figure 1). This study received ethical approval from the Human Research Institutional Review Board at Chang Gung Memorial Hospital, with approval ID 202002095B0 and an approval date of 8 December 2020.

### 2.1. Clinical and Laboratory Measurements

All patients underwent a comprehensive evaluation administered by proficient neurologists upon their enrollment. This evaluation encompassed exhaustive neurological and physical examinations, incorporating baseline clinical and laboratory assessments. The data collection process encompassed a range of parameters, including but not limited to age at disease onset, gender, height, waist circumference, body mass index (BMI), disease duration, systolic and diastolic blood pressures, and the presence of microvascular complications associated with diabetes. Furthermore, for each patient, the urinary albumin-to-creatinine ratio (UACR) [14] and the estimated glomerular filtration rate (eGFR) were computed, as previously documented [15].

### 2.2. Assessment of Cardiovascular Autonomic Functions

Each participant underwent a standardized assessment of cardiovascular autonomic function. Cardiac autonomic reflex tests (CARTs), recognized as the gold standard metrics for evaluating autonomic function in individuals with DM [5], were employed. Parameters derived from Ewing’s methods, such as heart rate responses to deep breathing (E:I ratio), heart rate responses to standing (30:15 ratio), heart rate responses to the Valsalva maneuver, and blood pressure responses to standing [16], were frequently employed by clinicians specializing in diabetes care.

### 2.3. Assessment of Cardiovascular Autonomic Neuropathy Severity

In our investigation, the severity of CAN was evaluated using the cardiovagal and adrenergic sub-scores derived from the CASS [17]. The assessment battery included tests measuring the heart rate response to deep breathing (HR_DB), Valsalva ratio (VR), and a 5 min head-up tilt (HUT) test, as outlined in Low’s methodology [6]. The specific techniques for calculating the HR_DB and VR were adopted from a previously published study [6]. In this particular study, the CASS employed a scale ranging from 0 to 7 points [18].

### 2.4. Measurements of Biomarkers for Chemerin, Oxidative Stress and Endothelial Dysfunction

The serum thiobarbituric acid-reactive substance (TBARS) levels were quantified to assess oxidative stress, while the serum total reduced thiols were measured to evaluate the antioxidative capacity in response to heightened oxidative damage. The determination of the serum TBARS and thiol levels was carried out using a commercially available assay kit (Cayman Chemical, Ann Arbor, MI, USA, cat. no. 10009055), following the prescribed guidelines provided by the manufacturer [19]. The serum levels of intercellular adhesion molecule 1 (sICAM-1), serum vascular adhesion molecule 1 (sVCAM-1), and chemerin measurements were determined using enzyme-linked immunosorbent assay (ELISA) kits commercially sourced from R&D Systems, Minneapolis, MN, USA, in strict adherence to the manufacturer’s provided instructions.

### 2.5. Sample Size Calculation

The sample size was estimated to have an effect size f^2^ of 0.15, which was determined using G*Power software version 3.1.9.2 (Universität Düsseldorf, Germany). Two predictors with the level of significance were set at α = 0.05. For the statistical power, 1 − β was set to 0.8, and the corr among the rep measures was set to 0.5. The total sample size was 68 using multiple linear regression: R^2^ deviation from zero. Assuming a dropout rate of 20%, a final total sample size of 82 was calculated.

### 2.6. Statistical Analysis

**Figure 2:**
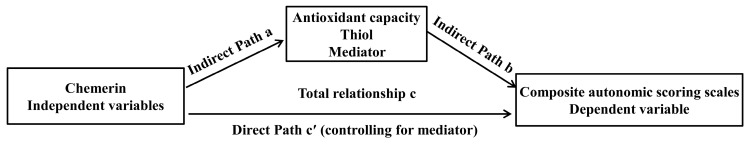
Diagram of the mediation hypothesis framework.

Data were presented as the mean ± SD or median (interquartile range). Continuous variables between the T2DM and prediabetes groups were compared with the independent t-test, while categorical data were compared using the chi-square or Fisher’s exact test. Non-normally distributed continuous variables were logarithmically transformed for the independent t-test. Correlation analysis assessed the relationships between the CASS values and baseline factors and biomarkers. Multiple linear regression determined the influence of variables correlated with the mean CASS value. A mediation model examined the causal links between chemerin (independent), thiol level (mediating), and CAN severity (CASS, dependent) (Figure 2). The Sobel test checked the significance of mediating effects [20]. All analyses were conducted using SPSS software (v26, IBM, Armonk, NY, USA).

## 3. Results

### 3.1. Baseline Characteristics of the Patients

Among the cohort of 184 patients, 143 individuals were diagnosed with T2DM, with a mean age of 70.3 years, while the remaining 40 patients exhibited prediabetic conditions, with a mean age of 65.8 years. The baseline characteristics, underlying medical conditions, various cardiometabolic parameters and macrovascular (coronary heart disease, cerebrovascular events, and peripheral artery disease) and microvascular complications (diabetic kidney disease, retinopathy, diabetic sensorimotor polyneuropathy, and CAN) in the T2DM and prediabetes patients are comprehensively detailed in Table 1. Patients with T2DM had significantly higher prevalence rates of diabetic kidney disease (*p* < 0.0001), diabetic sensorimotor polyneuropathy (*p* < 0.0001), and CAN (*p* = 0.005) compared to those with prediabetes. Notably, hypertension emerged as the predominant underlying ailment within both patient groups, closely followed by hyperlipidemia.

Comparative analysis revealed several noteworthy distinctions between the two groups. Patients afflicted with T2DM exhibited an advanced chronological age (*p* = 0.003) and displayed higher values for the BMI and waist circumference (*p* = 0.004 and *p* = 0.002, respectively). Conversely, this group demonstrated a lower prevalence of concomitant hyperlipidemia (*p* = 0.03). Furthermore, the baseline assessments of the peripheral blood parameters, as well as the levels of biomarkers encompassing oxidative stress, endothelial dysfunction, and chemerin, are meticulously outlined in Table 1. Patients diagnosed with T2DM were observed to possess lower concentrations of total cholesterol and LDL (*p* < 0.0001 and *p* = 0.013, respectively). Conversely, these individuals exhibited elevated values for the HbA1c (%), UACR, chemerin, TBARS, and sVCAM-1 levels (*p* < 0.0001, *p* < 0.0001, *p* < 0.0001, *p* = 0.002, and *p* = 0.006, respectively).

### 3.2. Baseline Assessment of Cardiovascular Autonomic Function in Patients

The baseline cardiovascular autonomic assessments of patients within the T2DM and prediabetes groups are comprehensively presented in Table 1. Notably, with the exception of the change observed between the minimum systolic blood pressure during the head-up tilt and the baseline systolic blood pressure, which did not exhibit statistical significance, all the other cardiovascular autonomic parameters demonstrated statistically significant differences. Specifically, the CASS values in the T2DM group exhibited a higher magnitude (*p* = 0.001), whereas the HR_DB, VR, E:I ratio, and the 30/15 ratio were found to be significantly lower within the T2DM cohort (*p* < 0.0001, *p* < 0.0001, *p* < 0.0001, and *p* = 0.03, respectively).

### 3.3. Influence of Biomarkers of Oxidative Stress, Endothelial Dysfunction, and Cardiometabolic Risk Factors on Composite Autonomic Scoring Scale Values and Chemerin Levels

Supplementary Table S1 provides the outcomes of a correlation analysis aimed at assessing the influence of biomarkers associated with oxidative stress, endothelial dysfunction, and cardiometabolic risk factors on the CASS values and chemerin levels. The statistical results, including the correlation coefficients and associated *p*-values, revealed notable associations as follows: the diabetes duration (r = 0.38, *p* < 0.0001), waist circumference (r = 0.22, *p* = 0.004), index HbA1c (r = 0.21, *p* = 0.005), eGFR (r = −0.35, *p* < 0.0001), UACR (r = 0.32, *p* < 0.0001), chemerin (r = 0.47, *p* < 0.0001), sVCAM-1 (r = 0.32, *p* = 0.002), TBARS (r = 0.31, *p* = 0.002), and thiols (r = −0.38, *p* < 0.0001) exhibited significant correlations with the CASS values. Additionally, the BMI (r = 0.27, *p* = 0.0006), waist circumference (r = 0.28, *p* = 0.005), eGFR (r = −0.49, *p* < 0.0001), UACR (r = 0.37, *p* < 0.0001), sVCAM-1 (r = 0.28, *p* = 0.004), TBARS (r = 0.41, *p* < 0.0001), thiols (r = −0.20, *p* = 0.04), triglyceride levels (r = 0.41, *p* < 0.0001), and high-density lipoprotein (HDL) levels (r = −0.21, *p* = 0.03) were also significantly correlated with the chemerin levels.

### 3.4. Associations of Significantly Influential Parameters with Composite Autonomic Scoring Scale Values

The impacts of various risk factors on the CASS values in the patients, as determined through correlation analysis, are presented in Supplementary Table S2. Our correlation analysis (Supplementary Table S1) revealed that several variables, including diabetes duration, waist circumference, index HbA1c, eGFR, UACR, chemerin, sVCAM-1, TBARS, and thiols, displayed statistically significant associations with the CASS values. To further discern the critical determinants contributing to the elevated CASS values, we employed a multiple linear regression analysis utilizing a stepwise procedure. This regression analysis identified free-form chemerin and thiols as independent variables significantly associated with the mean CASS value.

### 3.5. Mediation Analysis of Chemerin Levels, Antioxidant Capacity, and the Severity of Cardiovascular Autonomic Neuropathy

The primary hypothesis examined whether the impact of chemerin levels (independent variable) on the severity of CAN measured using the CASS, dependent variable) was indirectly explained by antioxidant capacity (thiol, mediator), accounting for a significant group main effect. The path model concurrently evaluated three critical effects: (a) the influence of the independent variable (chemerin) on the mediator (thiol) (indirect effect, path a); (b) the influence of the mediator on the dependent variable (CASS) (indirect effect, path b); and (c) the mediation effect (a × b), signifying the reduction in the association between the independent and dependent variables (total relationship, path c) when incorporating the mediator into the model (direct path, path c’). Simplified reporting revealed that the results met all three aforementioned criteria, indicating a significant mediating relationship (*p* = 0.04, Sobel test) (Figure 2, Supplementary Table S3).

## 4. Discussion

### 4.1. Major Findings of Our Study

In accordance with our initial hypothesis and consistent with the existing literature, individuals diagnosed with T2DM and prediabetes exhibited heightened levels of oxidative stress, reduced antioxidant capacity, elevated chemerin concentrations, and compromised cardiovascular autonomic function. Notably, our study observed effective control of various vascular risk factors, encompassing blood pressure, blood glucose, and lipid profiles, with the exception of central obesity—a modifiable risk factor that remained unmitigated in our investigation. Furthermore, our findings substantiate the notion that chemerin plays a contributory role in the exacerbation of CAN in individuals with well-regulated T2DM and prediabetes. Beyond chemerin, our results suggest that oxidative stress and endothelial dysfunction may also play significant roles in driving this pathological process.

### 4.2. Pathogenesis of Chemerin, Oxidative Stress, and Endothelial Dysfunction

The accelerated enlargement of the adipocyte dimensions among obese individuals occurs in a non-synchronized fashion, thereby resulting in a perturbation in the secretion of adipocytokines, particularly characterized by the elevated expression of pro-inflammatory adipocytokines [21]. This phenomenon is concurrently associated with compromised angiogenic processes, endothelial dysfunction, and the onset of microvascular complications [22]. Oxidative stress denotes a state characterized by an imbalance between the generation of reactive oxygen species and the capacity of the body’s antioxidant defense system to counteract them [23]. Our investigation revealed notably elevated oxidative stress levels (measured by TBARS level) and a discernible, although not statistically significant, reduction in the antioxidative capacity (measured by thiol levels) among individuals with type 2 diabetes in comparison to those with prediabetes.

Chemerin has been characterized as a multifunctional molecule, playing roles as a chemokine, adipokine, paracrine/autocrine agent, and growth factor [24]. Specifically, as a chemokine, chemerin contributes to chemoattraction within the vasculature, modifies endothelial adhesion levels, and undergoes extracellular activation within the vascular lumen [24] as well as in the initiation of immune responses by regulating leukocyte recruitment toward the site of inflammation [25]. In its role as an adipokine, chemerin influences lipid metabolism [26] and glucose regulation, potentially affecting their infiltration into the endothelium. Moreover, as a growth factor, chemerin promotes the growth of microvessels to support adipocytes and exerts an impact on osteoblast genesis. The findings of our study revealed a positive correlation between chemerin levels and heightened oxidative stress (as indicated by TBARs), diminished antioxidative capacity (measured by thiol levels), and increased endothelial dysfunction (reflected in sVCAM-1 concentrations). Additionally, our investigation demonstrated a positive association between chemerin levels and biomarkers indicative of microvascular complications in diabetes, such as diabetic kidney diseases and CAN. Consistent with prior research [27], our study also identified a positive correlation between chemerin and body mass index (BMI), while observing a negative correlation with high-density lipoproteins (HDLs).

### 4.3. Risk Factors Contributing to the Severity of Cardiovascular Autonomic Neuropathy

The pathophysiological underpinnings of CAN development are multifaceted, with several studies underscoring the pivotal role played by cardiovascular risk factors [3,28,29,30]. CAN manifests as a length-dependent pattern of malady, characterized by early-phase damage to parasympathetic activity and subsequent autonomic imbalance. With the progression of the disease, late-stage CAN is marked by sympathetic denervation [31]. Our study’s findings corroborated these observations, demonstrating significantly diminished parasympathetic parameters, including HR_DB, VR, E:I ratio, and 30:15 ratio, in patients with T2DM compared to those with prediabetes. However, the distinction in sympathetic parameters, as indicated by orthostatic blood pressure changes, did not exhibit pronounced differences between the two groups. These results align with prior investigations [32,33,34,35]. Notably, despite effective vascular risk factor management in our patient cohort, our study underscored the continued significance of chemerin levels in relation to the severity of CAN, as assessed using the CASS.

### 4.4. Association among Chemerin, Antioxidant Capacity and Cardiac Autonomic Function in T2DM and Prediabetes

Historically, chemerin has been linked to the cardiovascular system, albeit indirectly. It was often considered a potential confounder in cardiovascular research when assessing oxidative stress markers. Mediation analysis, on the other hand, aims to establish causal relationships and can explore causal links between chemerin levels and cardiovascular autonomic neuropathy. Additionally, it can assess how reduced antioxidant capacity may explain the impact of chemerin exposure on autonomic neuropathy severity. In this study, we hypothesized that antioxidant capacity mediates the relationship between chemerin levels and autonomic neuropathy severity.

### 4.5. Study Limitations

This study is subject to four significant limitations. Firstly, existing clinical research robustly supports the efficacy of intensified multifactorial intervention, comprising the management of hyperglycemia, hypertension, dyslipidemia, and microalbuminuria, in mitigating the risk of CAN progression in individuals with T2DM [35]. Consequently, further longitudinal investigations are warranted to validate the potential impact of multifactorial intervention and effective obesity control on modulating chemerin levels, thereby offering insight into their prospective role in mitigating CAN progression in both T2DM and prediabetic populations. Secondly, our study has uncovered a concurrent impairment in autonomic and renal functions, associated with heightened chemerin levels. The potential therapeutic benefits of employing multiple antihypertensive agents and precise blood glucose management to halt renal complications and ameliorate CAN in individuals with T2DM and prediabetes constitute a vital area necessitating further exploration. Thirdly, in our prospective observational study, we assessed a group of consecutively enrolled individuals with well-managed T2DM, effectively handling cardiometabolic risk factors such as hypertension and hyperlipidemia. The study covered a 2-year duration for the enrolled patients. Because medication regimens could change over this period, it became challenging to evaluate the effects of drugs specifically. Consequently, no comparisons regarding the effects of individual drugs were undertaken. Finally, although TBARS have been analyzed for decades to determine oxidative stress, there are also more contemporary methods such as isoprostanes, peroxides, and antibodies against oxidized LDL. The same applies to the detection of antioxidants, where there are numerous methods for assessing antioxidant status or polyphenol methods, as well as biomarkers for endogenous antioxidants. This should be considered in future studies.

## 5. Conclusions

This study illustrates the concurrent presence of compromised cardiovascular function, elevated chemerin levels, and diminished antioxidant capacity in individuals with T2DM and prediabetic conditions. Importantly, it underscores the interrelated nature of these manifestations. The mediation analysis results offer a potential pathophysiological mechanism explaining how heightened chemerin levels and reduced antioxidant capacity detrimentally affect the severity of CAN. Given the chemerin levels’ role as an indicative marker of CAN severity, a longitudinal investigation is essential to confirm whether reducing chemerin levels through intensified multifactorial intervention can effectively attenuate CAN progression.

## Figures and Tables

**Table 1:** Fundamental demographic characteristics of the patient cohort.

	Prediabetes (n = 40)	T2DM (n = 143)	*p*-Value
Characteristics			
Age (year)	65.8 ± 10.4	70.3 ± 7.9	0.003 *
Sex (male/female)	22/18	79/64	0.98
Diabetes duration (year)	--	11.2 ± 8.7	
Body mass index	25.2 ± 3.0	27.2 ± 5.8	0.004 *
Waist circumference (cm)	88.6 ± 9.0	94.3 ± 12.3	0.002 *
SBP (mmHg)	134.2 ± 19.6	137.6 ± 19.5	0.33
DBP (mmHg)	77.2 ± 11.3	76.9 ± 12.7	0.91
Baseline underlying disease			
Hypertension (%)	26 (65.0%)	107 (74.8%)	0.22
Hyperlipidemia (%)	28 (70%)	73 (51.0%)	0.03 *
Coronary heart disease (%)	2 (5.0%)	11 (7.7%)	0.58
Cerebrovascular events (%)	6 (15%)	34 (23.8%)	0.21
Peripheral artery disease (%)	0 (0)	4 (2.8%)	0.58
Diabetic kidney disease, n (%)	2 (5%)	52 (36.4%)	<0.0001 *
Retinopathy, n (%)	18 (45%)	85 (59.4%)	0.09
Diabetic sensorimotor polyneuropathy, n (%)	2 (5%)	52 (36.4%)	<0.0001 *
Cardiovascular autonomic neuropathy, n (%)	11 (27.5%)	66 (46.2%)	0.005
Laboratory test findings			
Total cholesterol (mmol/L)	175.4 ± 24.9	156.5 ± 27.6	<0.0001 *
Triglyceride (mmol/L)	117.3 ± 44.1	131.5 ± 74.2	0.13
HDL-C (mmol/L)	53.6 ± 14.3	49.8 ± 18.1	0.23
LDL-C (mmol L)	98.0 ± 18.8	87.5 ± 34.0	0.013 *
Index HbA1c (%)	6.0 ± 0.3	7.3 ± 1.2	<0.0001 *
eGFR (mL/min/1.73 m^2^)	65.9 ± 18.0	63.6 ± 24.6	0.91
UACR (mg/g)	5.8 (4.2, 10.9)	20.1 (7.6, 75.1)	<0.0001 *
Chemerin, ng/mL	76.2 ± 15.1	98.5 ± 32.0	<0.0001 *
Biomarkers of oxidative stress			
TBARS (μmol/L)	14.9 ± 4.6	20.9 ± 7.0	0.002 *
Thiol (μmol/L)	1.06 ± 0.34	0.95 ± 0.35	0.25
Biomarkers of endothelial dysfunction			
sICAM-1 (ng/mL)	214.9 ± 92.5	208.2 ± 70.3	0.75
sVCAM-1 (ng/mL)	617.5 ± 143.7	805.9 ± 250.2	0.006 *
Cardiovascular autonomic study			
CASS	0.80 ± 0.54	1.93 ± 1.48	0.001 *
HR_DB (beats/min)	11.56 ± 6.53	7.63 ± 5.23	<0.0001 *
Valsalva ratio	1.42 ± 0.18	1.28 ± 0.16	<0.0001 *
Delta SBP	−4.0 (−10.5, 1.0)	−5.0 (−13.0, 2.0)	0.96
E:I ratio	1.20 ± 0.11	1.12 ± 0.09	<0.0001 *
30/15 ratio	1.08 ± 0.06	1.06 ± 0.04	0.03 *

* Indicates that *p* value < 0.05. Data are presented as means ± standard deviations or medians (IQR). Abbreviations: n, number of cases; IQR, interquartile range, SBP, systolic blood pressure; DBP, diastolic blood pressure; eGFR, estimated glomerular filtration rate; UACR, urine albumin–creatinine ratio; HDL-C, high-density lipoprotein cholesterol; LDL-C, low-density lipoprotein cholesterol; TBARS, thiobarbituric acid-reactive substance; sICAM-1, serum intercellular adhesion molecule 1; sVCAM-1, serum vascular adhesion molecule 1; TBARS, thiobarbituric acid-reactive substance; CASS, composite autonomic scoring scale; HR_DB, heart rate response to deep breathing; CAN, cardiac autonomic neuropathy; Delta SBP, the change between the minimum systolic blood pressure during head-up tilt and baseline systolic blood pressure.

## Data Availability

The data from this study can be acquired from the corresponding author upon reasonable request.

## Supplementary Materials

The following supporting information can be downloaded at https://www.mdpi.com/article/10.3390/biomedicines11113024/s1, Table S1: Correlation analysis of oxidative stress, endothelial dysfunction and cardiometabolic parameters on the com-posite autonomic scoring scale values and chemerin levels; Table S2: Effects of the variables on the composite autonomic scoring scale values in patients according to the correlation analysis; and Table S3: A simple mediation model of chemerin ([X]) on the severity of cardiovascular autonomic neuropathy (composite autonomic scoring scale values [Y]) through oxidative stress (Thiol thiol [M]) effort.
