# Supplementary material for: Impacts of Chemerin Levels and Antioxidant Capacity on the Severity of Cardiovascular Autonomic Neuropathy in Patients with Type 2 Diabetes and Prediabetes
Source: Biomedicines. 2023 Nov 10;11(11):3024. doi: 10.3390/biomedicines11113024 (PMC10668959; doi:10.3390/biomedicines11113024)
Supplement: Supplementary file 1 [file biomedicines-11-03024-s001.zip › biomedicines-2703397-supplementary.pdf]

biomedicines MDPI

Supplementary Table S1. Correlation analysis of oxidative stress, endothelial dysfunction and cardiometabolic parameters on the composite autonomic scoring scale values and chemerin levels.

	Composite autonomic scoring scale		Chemerin (μg/mL)	
Variables	r	P value	r	P value
Age (year)	0.05	0.50	-0.14	0.16
Diabetes duration	0.38	<0.0001*	0.13	0.21
Body mass index	0.11	0.14	0.27	0.006*
Waist circumstance (cm)	0.22	0.004*	0.28	0.005*
Index HbA1c (%)	0.21	0.005*	0.10	0.30
eGFR (mL/min/1.73 m^2)	-0.35	<0.0001*	-0.49	<0.0001*
UACR (mg/g)	0.32	<0.0001*	0.37	<0.0001*
Chemerin (ng/mL)	0.47	<0.0001*	--	--
sICAM-1 (ng/mL)	0.03	0.81	0.14	0.14
sVCAM-1 (ng/mL)	0.32	0.002*	0.28	0.004*
TBARS, μmol/L	0.31	0.002*	0.41	<0.0001*
Thiols, μmol/L	-0.38	<0.0001*	-0.20	0.04*
Total cholesterol(mmol/L)	-0.08	0.28	0.11	0.26
Triglyceride(mmol/L)	0.04	0.65	0.41	<0.0001*
HDL-C (mmol/L)	-0.001	0.99	-0.21	0.03*
LDL-C (mmol/L)	-0.05	0.5	0.15	0.13

r: correlation coefficient. * Indicates that p-value <0.05. Abbreviations: HDL-C, high-density lipoprotein cholesterol; LDL-C, low-density lipoprotein cholesterol; UA, uric acid; HbA1c, glycohemoglobin; eGFR, estimated glomerular filtration rate; UACR, urine albumin-creatinine ratio; TBARS, thiobarbituric acid-reactive substance; sICAM-1, serum intercellular adhesion molecule 1; sVCAM-1, serum vascular adhesion molecule 1

Supplementary Table S2. Effects of the variables on the composite autonomic scoring scale values in patients according to the correlation analysis.

	Model		
	Regression coefficient	Standard error	P value
Constant	0.56	0.94	0.56
Chemerin, ng/mL	5.04	2.65	0.035*
Thiol, μmol/L	0.17	0.06	0.007*

Model: R^2=0.418. Predictors in the model: constant, chemerin, thiol; * Indicates that p-value <0.05

Supplementary Table S3. A simple mediation model of chemerin ([X]) on the severity of cardiovascular autonomic neuropathy (composite autonomic scoring scale values [Y]) through oxidative stress (thiol [M]) effort.

	Path coefficient	Standard error	P-value
**Total effects (total relationship, path c) Ω**			
The relationship between the chemerin (independent variable) and CASS (dependent variable)	0.024	0.004	<0.0001*
**Direct effects, path c′**			
The relationship between the chemerin (independent variable) and CASS (dependent variables) by including the thiol (mediator) into the model	0.021	0.004	<0.0001*
**Indirect effect, path a**			
The effect of the chemerin (independent variable) on the thiol (mediator)	-0.002	0.001	0.038*
**Indirect effect, path b**			
The effect of the thiol (mediator) on the CASS (dependent variable) by controlling the effect for the chemerin (independent variable)	-1.472	0.406	<0.0001*

Abbreviations: X, chemerin (independent variable); Y, severity of cardiovascular autonomic neuropathy (CASS) (dependent variable); M, thiol (mediator); * Indicates that p value <0.05

Ω = the mediation effect a × b, which is defined as the reduction in the relationship between the independent and dependent variables (chemerin severity of peripheral nerve dysfunction) (total relationship, path c) by including the mediator into the model (direct path, path c′); (Sobel test, P=0.04)
